# Effect of sevoflurane preconditioning on light-induced retinal damage in diabetic rats

**Published:** 2018

**Authors:** Daniela Adriana Iliescu, Alexandra Ciubotaru, Mihai Aurelian Ghiţă, Adrian Dumitru, Leon Zăgrean

**Affiliations:** * Physiology Department, “Carol Davila” University of Medicine and Pharmacy, Bucharest, Romania; ** Pathology Department, University Emergency Hospital, Bucharest, Romania; *** Ophthalmology Department, “Dr. Carol Davila” Central Military University Emergency Hospital, Bucharest, Romania; **** Ophthalmology Department, University Emergency Hospital, Bucharest, Romania

**Keywords:** diabetes, photostress, preconditioning, sevoflurane

## Abstract

Hyperglycemia and bright light are powerful stress agents that produce an enhanced retinal damage, when simultaneously acting on retina. Previous studies have shown that preconditioning with sevoflurane anesthesia offers a certain degree of protection to retinal cells against light damage. The objective of this study was to explore the effect of sevoflurane anesthetic preconditioning on a model of light-induced retinal degeneration in diabetic rats. Wistar rats that were randomly divided into four groups: control (rats exposed to photostress), group 1 (rats exposed to photostress and sevoflurane preconditioning), group 2 (diabetic rats exposed to photostress), group 3 (diabetic rats exposed to photostress and sevoflurane preconditioning) were used for this experiment. We recorded basal electroretinogram (ERG), at 36 h and 14 days after photostress and performed histological analysis of the retina. Results showed that sevoflurane has a protective effect on light-induced neuroretinal degeneration proved by significantly less variations of the ERG before and after photostress. Diabetes appears to increase the damaging effect of photostress on retina and attenuate the protection provided by sevoflurane preconditioning.

## Introduction

Light induced retinal injury is an important iatrogenic complication that can occur during ophthalmologic surgery. In order to prevent or attenuate it, strategies to protect the retina should be developed. The main purpose of this study was to explore the effect of sevoflurane anesthesia preconditioning on a model of light-induced retinal degeneration in rats and to determine if sevoflurane has a neuroprotective role for retinal cells. In order to prove our theory, we further increased the stress load on the retina, by inducing diabetes to rats and subjecting them to the same protocol.

Light induced retinal injury: Clinical context

The eye has many physiological defensive mechanisms against light damage, for example: squint, blink and miosis reflexes, the cornea reflecting most of the non-incidental light and absorbing UV-B, UV-C, furthermore, the lens absorbing most of the UV-A and some of the near infrared. The light absorption process is also presented in the other eye optical media, but to a lesser extent [**1**]. 

Most of the inherent protection mechanisms of the eye are abolished during cataract, refractive, corneal transplant, and above all vitreoretinal surgery. Although retina has its own additional defenses, high power, and prolonged radiation from very close sources, like operating microscope or fiber optic endoilluminator, can cause serious photic lesions [**2**]. A light induced retinal injury is first detected by fluorophotometry, due to a transitory blood-retinal barrier dysfunction, an area of retinal edema appearing after 1-2 days, followed by a focal hyperpigmentation of RPE (retinal pigment epithelium), ophtalmoscopically observable, after 1 week [**3**]. 

It is noteworthy that retinal phototoxicity exists without clinically visible lesions. Studies have shown a statistically significant reduction of visual acuity in patients operated under higher light intensity in comparison with those operated under lower light intensity [**4**]. 

Light induced retinal injury: Physiopathological mechanisms

Like radiation, light can damage tissues through ablation, overheating, and phototoxicity, depending on frequency spectrum and power. In our study, we were mostly interested in phototoxicity, i.e. the processes triggered by intense light exposure that could eventually promote an apoptotic cell death in photoreceptors and other retinal cells. We intended to interfere with phototoxicity, attenuating and preventing its damaging consequences. Phototoxicity comprises multiple pathological mechanisms, such as light-induced oxidative reactions, toxic photoproducts generated by vitamin A exposure to light and metabolic abnormalities [**5**]. 

Taking into consideration that our study was made on Wistar rats, which have a rod-dominant retina, we can attribute a central role in photoreceptor injury to rhodopsin bleaching [**6**]. The lack of rhodopsin in KO mice, or low levels of proteins involved in its regeneration, e.g. RPE-65, prevent light damage [**5**]. A human rod has approximately 4x107 rhodopsin molecules [**7**] and other mammals have a similar order of magnitude. Hence, from the light absorption and implicit energy perspective, it is an important aspect that we will integrate later in the context of oxidative stress. On the other hand, traces of all-trans- and 9-cis retinoic acid have been found in dark reared rat retinas exposed to intense light [**8**]. Knowing that retinoids are transcription regulators during development and mediate retinal cell differentiation and apoptosis [**9**] and that retinoic acid receptor and retinoid X receptor are expressed in various cell types in mature retina, including photoreceptors, might suggest a link between light exposure and transcriptional events. 

Close metabolic and anatomical relationships between neuroretina and RPE link damage in one tissue to degeneration in the other. Light induced reactive oxygen species lead to: cytotoxic oxysterols formation [**10**], protein coupling with other unsaturated lipids oxidation products, that impair several metabolic functions [**11**] and eventually, shading of this photoproducts along with rod outer segment (ROS) tips, followed by phagocytosis inside RPE and finally inducing RPE toxicity. Chromophores, such as rhodopsin bleaching products, RPE residues like A2E (bis-retinaldehyde-phosphatidylethanolamine) and other retinal containing molecules, induce retinal damage by increasing the likelihood of high-energy photons absorption and consequent oxidative stress [**12**]. Another strong argument, for oxidative stress involvement, is that treatment with antioxidants like ascorbic acid reduces phototoxicity [**13**,**14**] and promoters of glutathione (GSH) synthesis attenuate oxidative stress and decrease cell death [**15**].

A metabolic survey, made on rat retinas exposed to mild photostress, highlighted an increase in amino acids and biogenic amines specific to nitric oxide (NO) pathway [**16**]. Another previously made study gave NO a central role in mouse rods apoptosis induced by acute light damage. Specific inhibition of neuronal isoform of nitric oxide synthase (nNOS), not only prevents apoptosis, but it also blocks its early photoreceptor specific signs, like intracellular Ca2+ concentration increase. The postulated downstream pathway involves enhancement of the guanylate cyclase activity by NO, subsequent raised intracellular cGMP concentration, followed by cationic cGMP-gated channels opening and Ca2+ influx, which furthers the apoptotic program through mitochondrial membrane depolarization and apoptotic promoters realizing. The authors additionally suggested the possibility of Ca2+ dependent endonuclease activation [**17**].

**Sevoflurane preconditioning**

In general, preconditioning is a method through which prior subjection to certain stimuli can induce ulterior protection against a subsequent stressor. Volatile anesthetics have a long history as preconditionants, firstly used in cardiology against myocardial ischemic injury and then in neurology against ischemic and reperfusion injury [**18**-**20**].

Retinal preconditioning history is also starting to take shape [**21**-**27**] and we decided to explore the yet unknown potential of sevoflurane as a shield against photostress. Literature presents both ischemia and light as preconditioning agents for retina and not only stressors. But their protective mechanisms involve synthesis of neurotrophic molecules like basic fibroblast growth factor (bFGF) and ciliary neurotrophic factor (CNF), for light preconditioning [**21**] and Heat-shock protein 27 (Hsp 27), for ischemic preconditioning [**28**]. At the same time, sevoflurane acts directly on the existing receptors, modulating the cells activity more promptly.

With a number of advantages over isoflurane, sevoflurane is gaining popularity in clinical practice. The already existing infrastructure would be the upper hand in instituting preconditioning therapy with sevoflurane before ophthalmologic surgery, if proven efficient. A meta-analysis split the neuroprotective effects of sevoflurane and other inhalational anesthetics in three main paradigms: anti-excitotoxicity, anti-inflammation, and anti-apoptosis [**29**]. Sevoflurane diminishes neuron excitotoxicity by attenuating excitation and enhancing inhibition, i.e. it is an antagonist for ionotropic glutamate receptor and a positive modulator for GABA-A receptor [**30**,**31**]. Inflammation is as well present in light damage, so the fact that sevoflurane suppresses the expression of inflammatory cytokines, NF-kappa B and p38 MAPK is promising [**20**]. Another encouraging evidence is the up-regulation of antioxidant enzymes in rat brain following sevoflurane treatment [**32**], especially knowing that reactive oxygen species and oxidative stress play an important role in light induced retinal damage [**13**,**14**].

**Diabetes as a retinal stress load increase**

Apart from classic physiopathological mechanisms involved in diabetic retinopathy, such as endothelial tight junction disassembly, that leads to increase in vascular permeability and leukostasis, known as glucose-induced microvascular disease [**33**], important roles are played by oxidative stress and inflammation [**34**], both present in photic injury. The coexistence of light stress and diabetes mellitus was recently proved to potentiate retinal damage [**35**]. The increment in basal oxidative stress is due to the decrease of intracellular antioxidant glutathione (GSH). GSH biosynthesis is dependent on NADPH as a hydrogen donor. At the same time, glucose excess is converted to sorbitol by aldose reductase, an enzyme that also uses NADPH as a cofactor. Since retinal glucose uptake is insulin-independent, it increases significantly when the blood levels are high. The excess is converted to sorbitol, hence the diabetic retina sensibility to oxidative stress [**36**]. Furthermore, several inflammation markers are present in diabetic retinopathy: microglia activated morphology and proinflammatory cytokines secretion, elevated complement levels, increased expression of growth factors, including vascular endothelial growth factor (VEGF) [**34**].

Taking into consideration both the physiopathology and the high prevalence of diabetes mellitus, 8.5% in the adult population in 2014, we concurred that diabetes would be a suitable additional stressor to test our theory and to gain insight into the interaction between preconditioning, photostress, and diabetes. 

## Materials and methods

20 male Wistar rats, weighting 350 g and of approximately 3 months old were used in our study, which were reared in a normal 12h light/ dark cycle, with food and water ad libitum. Rats were divided equally and randomly into four groups: control (rats exposed to photostress), group 1 (rats exposed to photostress and sevoflurane preconditioning), group 2 (diabetic rats exposed to photostress) and group 3 (diabetic rats exposed to photostress and sevoflurane preconditioning). For ERG recording, we made use of electrodes, positioned as it follows: the active electrode on the stimulated eye, the reference on the mouth and the ground on the tail. The tips of the electrodes were made from nickel-chromium Alloy (ni80cr20, diameter 0.15 mm). Before ERG recording, we instillated oxibuprocaine hydrochloride (Benoxi 4 mg/ ml, UnimedPharma), a corneal anesthetic, and tropicamide (Mydriacyl, 0.5%, Alcon). For the ERG set-up, we connected the electrodes to Biopac MP150 System. We used Acq.Knowledge 4.2 for data acquisition and analysis. We recorded ERG for a 200 s period and waveforms were acquired by averaging 150 ms of the raw data after light stimulus. All recordings were done in a dark room after proper dark adaptation. The ERG recordings were done under anesthesia with chloral hydrate (0.4 g/ kg) injected intraperitoneally.

Photostress was performed with previous pupillary dilation with tropicamide (Mydriacyl, 0.5%, Alcon). For light exposure, we used an aluminium foil covered box, which contained four LED lamps that produced a mean brightness of 20.000 lux. The box was provided with a cooling system to prevent overheating. We monitored the temperature inside the box and ensured that the animals had the eyes opened at every 20 minutes during the experiment. The exposure time to photostress for all groups was 1 hour. ERG were performed at 36 h and 14 days after photostress in all groups. ERG a- and b-waves latencies and amplitudes were compared between groups.

Sevoflurane preconditioning was obtained by 1h exposure to 2% inhalational concentration through a specially adapted mask, at an oxygen flow rate of 2L/ min. The photostress was performed at one hour after sevoflurane preconditioning to ensure awakening from anesthesia before light exposure. Diabetic groups were formed by an intraperitoneal injection of streptozotocin at a dose of 50 mg/ kg body weight, one month before ERG baseline recording. Glycemia was measured 2 days after streptozotocin injection and after every ERG recording. Only glycemic values over 400 mg/ dl were considered suitable.

After ERG recordings were done, animals were sacrificed with an overdose of chloral hydrate. Eyes were enucleated for histological assessment. After enucleation, the eyes were fixed in paraformaldehyde 4%. Retinal sections and staining with hematoxylin and eosin were done. Analysis of retinal layers was performed. For statistical analysis, parameters were compared by using the one-way ANOVA test (IBM SPSS Statistics 22).

**Table 1:** Sequence of events conducted in our study

Control group	Day 1 Baseline ERG recording	Day 7 Photostress Exposure	Day 8 ERG recording – 36h after photostress	Day 21 ERG recording – 14 days after photostress
Group 1	Baseline ERG recording	Photostress Exposure + sevoflurane preconditioning	ERG recording – 36h after photostress	ERG recording – 14 days after photostress
Group 2 (diabetic)	Baseline ERG recording	Photostress exposure	ERG recording – 36h after photostress	ERG recording – 14 days after photostress
Group 3 (diabetic)	Baseline ERG recording	Photostress Exposure + sevoflurane preconditioning	ERG recording – 36h after photostress	ERG recording – 14 days after photostress

## Results

**Fig. 1:**
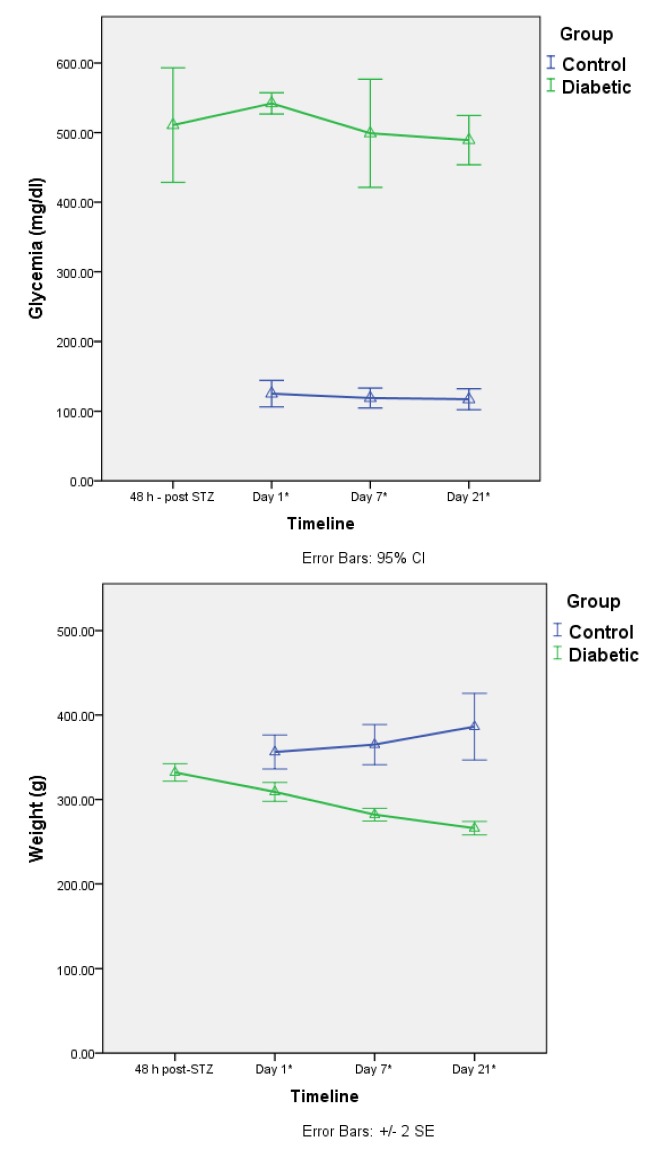
Glycemia and weight in control and diabetic animals. A mean glycemia of 500 mg/ dl and a decrease in weight over the course of the experiment in diabetic animals can be observed. *Day 1 represents the date of baseline ERG recording and corresponds to 4 weeks of streptozotocin (STZ) induced diabetes

We assessed the differences in ERG response between diabetic and non-diabetic groups, with or without prior sevoflurane preconditioning, after light-induced retinal damage. No statistical significant differences were found between the control group and the experimental groups at baseline measurement. ERG maintained the same measurements for a wave latency and b wave latency in the diabetic group (4 weeks after streptozotocin injection). Only the a-b amplitude showed a slight decrease, but it was not statistically significant.

**Fig. 2:**
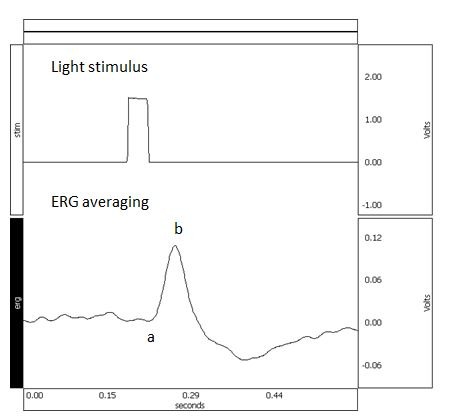
Baseline ERG averaging from one animal recording (specific morphology of the ERG waves)

**Fig. 3:**
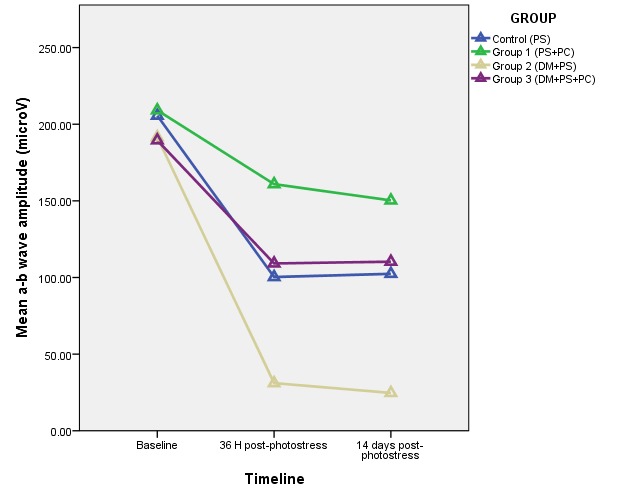
Variability of mean a-b wave amplitude in relation to sevoflurane preconditioning and diabetes after photostress (DM – diabetes mellitus, PS – photostress, PC – sevoflurane preconditioning)

**Fig. 4:**
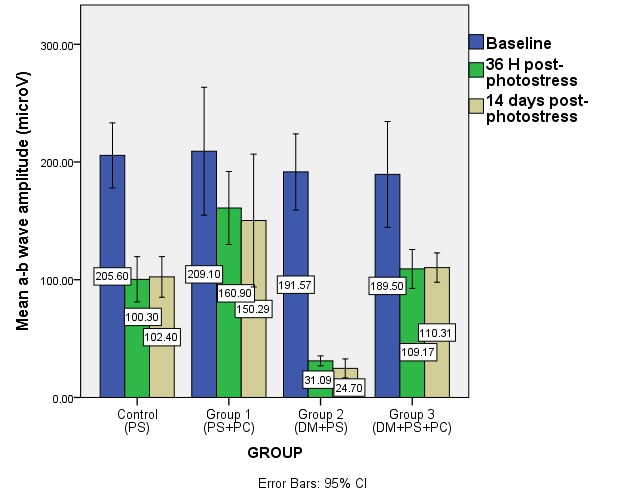
Mean a-b wave amplitude at baseline and after photostress in the studied groups (DM – diabetes mellitus, PS – photostress, PC – sevoflurane preconditioning)

The amplitude of the ERG showed a statistically significant decrease after photostress for both normal and diabetic animals (control and group 2). This decrease was more important in the diabetic group. For the photostress and preconditioning group (experimental group 1), the decrease was not statistically significant when baseline and post-photostress ERG were compared. Group 3 showed a statistically significant decrease of the ERG amplitude after photostress when compared to baseline. The mean amplitude values obtained after light damage were similar at 36 h and 14 days post-photostress for each of the studied groups.

**Fig. 5:**
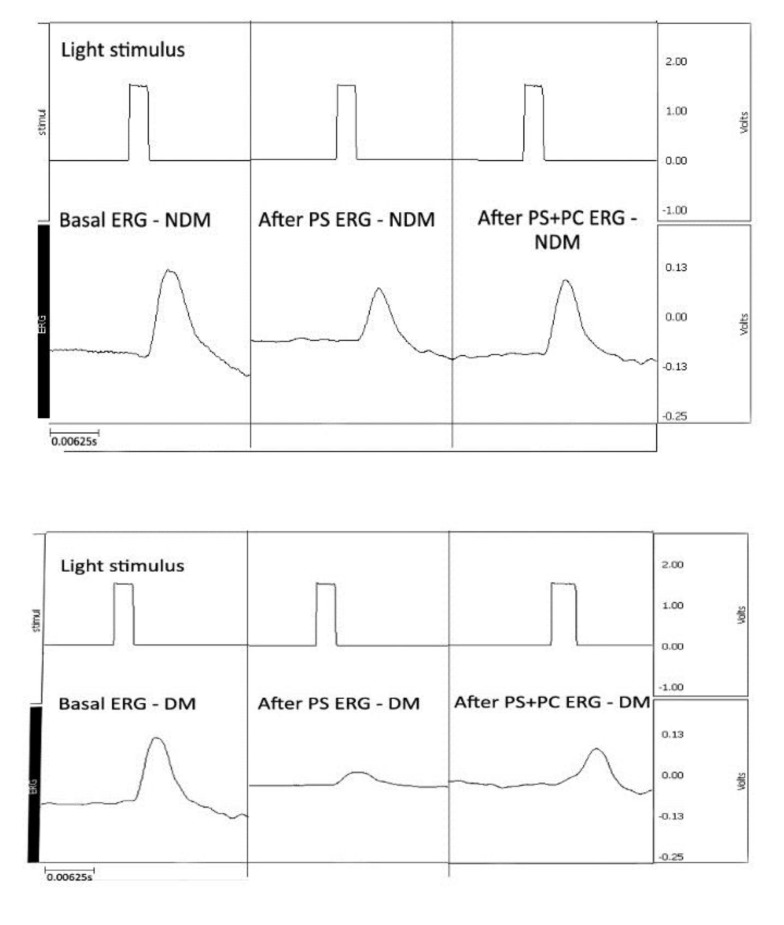
ERG recording at baseline, after photostress or photostress and sevoflurane preconditioning at 36 h (NDM – non-diabetic, DM - diabetes mellitus, PS–photostress, PC– sevoflurane preconditioning)

**Fig. 6:**
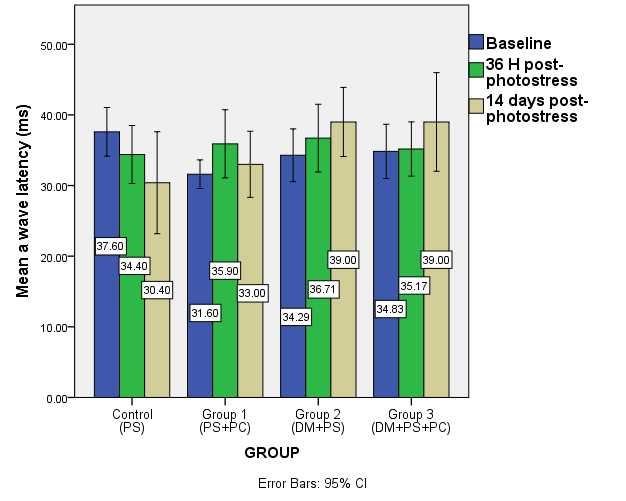
Mean a-wave latency at baseline and after photostress in the studied groups (DM – diabetes mellitus, PS – photostress, PC – sevoflurane preconditioning)

**Fig. 7:**
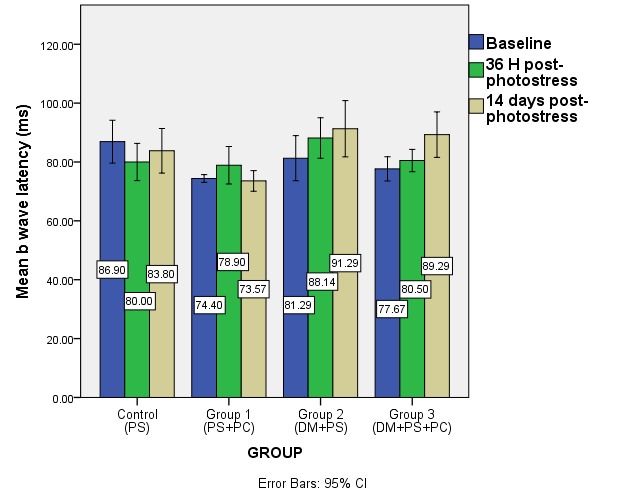
Mean b-wave latency at baseline and after photostress in the studied groups (DM – diabetes mellitus, PS – photostress, PC – sevoflurane preconditioning)

Both a- and b-wave latency showed no statistical changes after photostress or sevoflurane preconditioning and photostress in the non-diabetic groups at 36 h and 14 days after light damage. In the diabetic groups, the latencies of the composing ERG waves increased after 14 days post-photostress (not statistically significant), without additional shifts done by sevoflurane preconditioning.

**Fig. 8:**
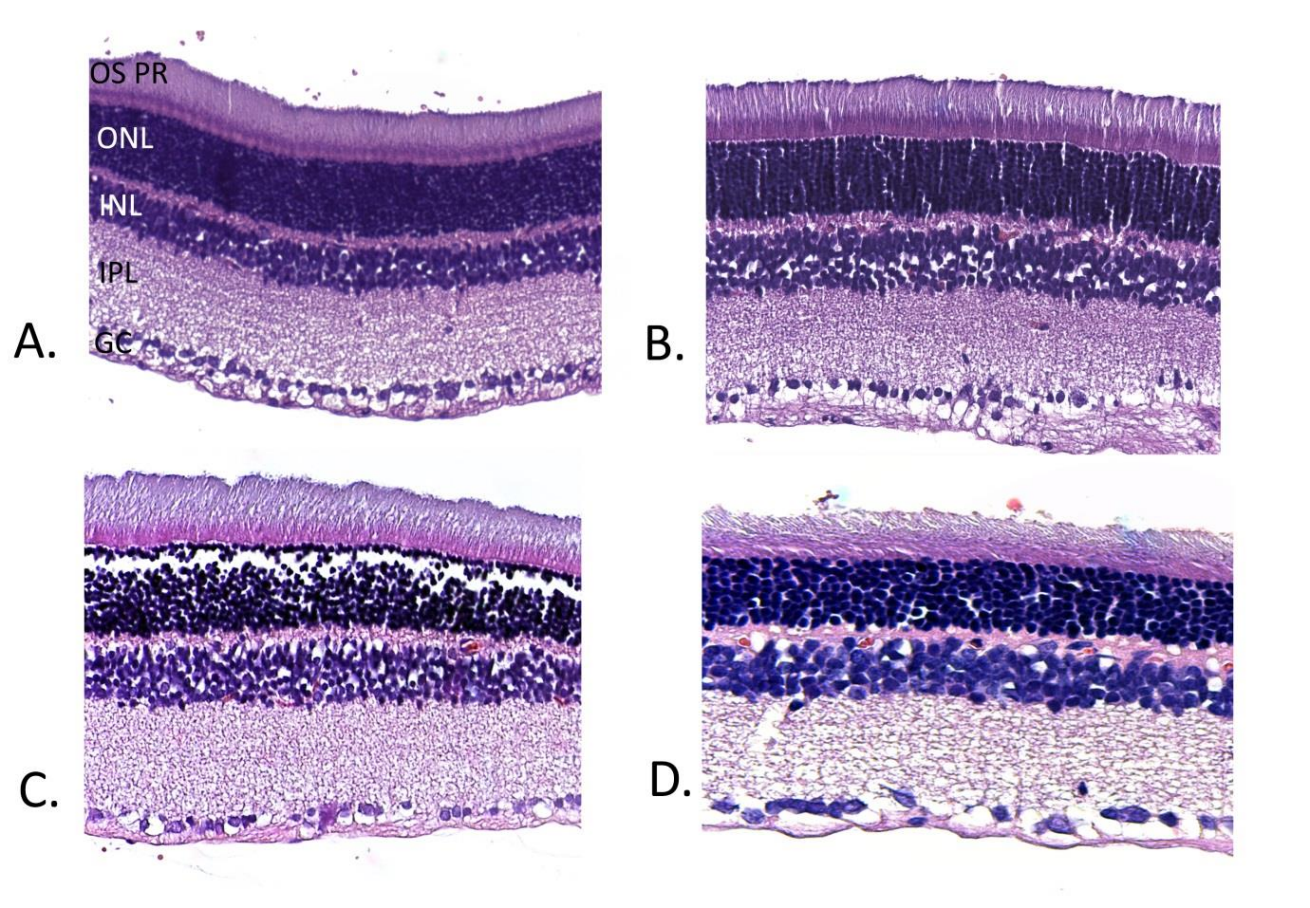
Hematoxylin & eosin staining of retinal layers in non-diabetic rats after photostress (A), non-diabetic rats after sevoflurane preconditioning and photostress (B), diabetic rats after photostress (C) and diabetic rats after sevoflurane preconditioning and photostress (D). In diabetic animals, sections reveal loss of retinal ganglion cell and a swollen appearance of the remaining cells. Additionally, the diabetic groups (C, D) show a decrease in cell density in ONL. A comparison between diabetic (D) and non-diabetic (B) groups, both with preconditioning and photostress, also exhibits a decrease in ONL thickness (OS PR – photoreceptor outer segment; ONL – outer nuclear layer; INL – inner nuclear layer; IPL – inner plexiform layer; GC – ganglion cell layer)

## Discussions

Our results showed that baseline ERG was not changed in any of the studied groups. This indicated that streptozotocin induced diabetic rats did not exhibit any ERG delay or decrease in amplitude by 4 weeks post-streptozotocin. Some studies have reported early visual function deficits in diabetic rats after 12 weeks following streptozotocin treatment [**37**] or even at one month following streptozotocin treatment [**38**]. On the other hand, electrophysiological studies done on female diabetic rats showed no visual function abnormalities after 12 weeks of streptozotocin-induced hyperglycemia [**39**]. Our study supported the fact that ERG was not modified after 4 weeks of streptozotocin induced diabetes in rats. A slight delay in a- and b-wave latency could be noted at 14 days post-photostress only in the diabetic groups but it did not reach statistical significance. This delay could be attributed to diabetes and not to photostress because it did not appear in the non-diabetic group after light damage.

The response to light induced damage was different in diabetic vs. control group. In the control group, our model of photostress produced a decrease to approximately half the mean amplitude value of the baseline ERG. This susceptibility to light damage is known to be much higher in albino rats, like Wistar rats, than in pigmented rodents [**40**]. Preconditioning with sevoflurane anesthesia provided a protective action on photoreceptors (represented by the a-wave of the ERG) and the bipolar cells (represented by the b-wave). This was indicated by the increased amplitude of the ERG after photostress when compared to control group. Positive effects of other anesthetics on protecting the retina against light-induced retinal damage have been described. Combined ketamine-xylazine and halothane anesthesia have been shown to be neuroprotective and reduce photoreceptor cell death [**41**,**42**]. Also sevoflurane has been recognized as having retinoprotective effects in rats by preconditioning in a model of retinal ischemia by permanent bilateral common artery occlusion [**25**].

Light-induced retinal damage produced a severe decrease of the a-b wave amplitude in the diabetic group indicating that diabetes is an additional stressor factor to photostress. This effect of summation between hyperglycemia in diabetes and light exposure interacts to induce a severe damage to retinal cells. This is consistent with other studies that have shown electrophysiological changes of ERG and VEP after photostress in diabetes type 1 without retinopathy [**43**]. Also histological analyses have shown reduced outer nuclear layer after a period of 9 days of light exposure in streptozotocin injected animals when compared to control group [**44**]. Other studies have reported that retinal deterioration due to white light was so severe in diabetic group after 42 days of light exposure to 1500-2000 mW/ m² during a 12 to 12 hour light-dark cycle, that ERG could not be recorded. Such severe light damage cannot be determined by other types of light, like red light, attenuated brown light, or attenuated yellow light [**35**].

Sevoflurane preconditioning with 2% inhalational concentration for 1 hour before photostress did provide some retinal protection against light-induced damage in diabetic animals but this effect was attenuated when compared to the non-diabetic group. The relationship between glycemia and anesthetic concentration has been speculated as an important determinant during anesthetic preconditioning in diabetes, higher concentrations of anesthetic having a more protective effect in diabetes [**45**]. Further studies should clarify if a higher inhalation concentration of sevoflurane (more than 2%) would offer a better retinal protection against light damage in diabetes. 

Beside anesthesia, other factors like ischemia conditioning have shown to provide some protection in diabetes [**46**,**47**]. This gives the “preconditioning” concept a launching pad for the development of new treatment strategies in diabetes. Nevertheless, other studies showed opposite results regarding diabetes and preconditioning [**48**].
